# Microsatellite instability in colorectal cancer and association with thymidylate synthase and dihydropyrimidine dehydrogenase expression

**DOI:** 10.1186/1471-2407-9-25

**Published:** 2009-01-20

**Authors:** Søren A Jensen, Ben Vainer, Mogens Kruhøffer, Jens B Sørensen

**Affiliations:** 1 Department of Oncology, Rigshospitalet, Copenhagen University Hospital, Copenhagen, Denmark; 2 Department of Pathology, Rigshospitalet, Copenhagen University Hospital, Copenhagen, Denmark; 3 Department of Clinical biochemistry, Skejby, Aarhus University Hospital, Aarhus, Denmark

## Abstract

**Background:**

Microsatellite instability (MSI) refers to mutations in short motifs of tandemly repeated nucleotides resulting from replication errors and deficient mismatch repair (MMR). Colorectal cancer with MSI has characteristic biology and chemosensitivity, however the molecular basis remains unclarified. The association of MSI and MMR status with outcome and with thymidylate synthase (TS) and dihydropyrimidine dehydrogenase (DPD) expression in colorectal cancer were evaluated.

**Methods:**

MSI in five reference loci, MMR enzymes (hMSH2, hMSH6, hMLH1 and hPMS2), thymidylate synthase (TS) and dihydropyrimidine dehydrogenase (DPD) expression were assessed in paraffin embedded tumor specimens, and associated with outcome in 340 consecutive patients completely resected for colorectal cancer stages II-IV and subsequently receiving adjuvant 5-fluorouracil therapy.

**Results:**

MSI was found in 43 (13.8%) tumors. Absence of repair protein expression was assessed in 52 (17.0%) tumors, which had primarily lost hMLH1 in 39 (12.7%), hMSH2 in 5 (1.6%), and hMSH6 in 8 (2.6%) tumors. In multivariate analysis MSI (instable) compared to MSS (stable) tumors were significantly associated with lower risk of recurrence (hazard ratio (HR) = 0.3; 95% CI: 0.2–0.7; P = 0.0007) and death (HR = 0.4; 95% CI: 0.2–0.9; P = 0.02) independently of the TS and DPD expressions. A direct relationship between MSI and TS intensity (P = 0.001) was found, while there was no significant association with DPD intensity (P = 0.1).

**Conclusion:**

The favourable outcome of MSI colorectal carcinomas is ascribed mainly to the tumor biology and to a lesser extent to antitumor response to 5-fluorouracil therapy. There is no evidence that differential TS or DPD expression may account for these outcome characteristics.

## Background

Colorectal cancer is the fourth most common malignant tumor in Western Europe and Northern America affecting 7% of the population and ranks as the second leading cause of cancer-related mortality [1].

The majority of colorectal cancers display aneuploidy appearing as chromosomal anomalies, whereas the remainder that constitutes 15–20% of these cancers is characterized by microsatellite instability (MSI) [2-6].

Microsatellites are DNA sequences in which a short motif of 1–5 nucleotides are tandemly repeated ten to hundred times. Microsatellites are prone to mutation during replication due to transient split of the two helical strands and slippage of the DNA polymerase complex at reannealing, which generate an insertion or deletion loop depending on slippage direction. Unless such mismatch is corrected, the loss or gain of repeated units on the daughter strand results in length variation termed microsatellite instability (MSI) [7].

The mismatch repair (MMR) is performed by the proteins hMSH2 heterodimerized with hMSH6 for recognition of a loop of few mismatched extrahelical nucleotides. Upon assemblage this complex interact with another heterodimeric complex, composed of hMLH1 and hPMS2 [8].

Deficient MMR that arise in sporadic colorectal cancer is nearly always due to an epigenetic biallelic hypermethylation of the hMLH1 gene promoter. In addition, MMR deficiency may result from genetic disorders, caused by an inherited germ-line mutation of one allele followed by an acquired alteration of the wild-type allele leading to inactivation of one of the three main MMR genes (MLH1, MSH2, and MSH6) [7].

While most of the half million microsatellite sequences scattered in the human genome are located within untranslated regions, in which MSI is assumed to be without significance to expression, a number of genes involved in mitosis and apoptosis carry microsatellites in their encoding regions [9]. MMR deficiency may promote malignant transformation as it allows accumulation of microsatellite insertion/deletion mutations, leading to MSI-driven inactivation of genes having key regulatory functions [10]. Besides being pathogenetic to malignant transformation MSI also characterises a subset of colorectal cancers with characteristic biology and chemosensitivity. Accordingly, in pooled analysis of patients with local and advanced disease high-frequency MSI (MSI-H) was associated with a favorable prognosis compared to microsatellite stable/low-frequency MSI (MSS/MSI-L) independently of chemotherapy [11]. Moreover, in the context of 5-fluorouracil therapy patients with MSS/MSI-L tumors had improved overall survival, whereas no similar benefit in outcome pertained to MSI-H tumors [2,12].

Various features of MMR deficient cancer cells as tolerance to accumulate 5-fluorouracil adducts and conspicuous lymfocytic infiltration in tumors have been put forward to account for the opposing trends of relative resistance of chemotherapeutic interventions, against the background of a favourable natural history [13].

Whether microsatellite instability deregulates genes related to tumor growth and response to 5-fluorouracil therapy, however, has not been clarified. Two such biomarkers thymidylate synthase (TS) [14-18] and dihydropyrimidine dehydrogenase (DPD) [19] play key roles for response to 5-fluorouracil therapy of colorectal cancer. The main mode of action is through irreversible inhibition of TS, whereas the major part of an administered 5-fluorouracil dose is catabolised by DPD. In addition, these enzymes may be considered prognostic for the outcome of colorectal cancer independently of chemotherapy as they regulate tumor pyrimidine homeostasis by catalyzing synthesis and degradation, respectively [14-22].

This retrospective study aimed to evaluate the association of MSI and MMR deficiency with outcome and with thymidylate synthase (TS) and dihydropyrimidine dehydrogenase (DPD) expression in tumors from 340 consecutive patients who were completely resected for colorectal cancer stage II-IV and subsequently received adjuvant 5-FU treatment.

## Methods

### Patients and chemotherapy

Consecutive patients completely resected for colon or rectal carcinomas stages II-IV, who received adjuvant chemotherapy at Department of Oncology, Rigshospitalet, Copenhagen University Hospital in the period February 1996 to December 2003 were included.

The adjuvant treatment was according to the Mayo regimen (Mayo Clinic, Rochester, MN), including bolus infusion of 5-fluorouracil (425 mg/m^2^) and isovorin (10 mg/m^2^) for 5 days, repeated every 4 weeks, for 6 courses. Data on clinical and pathological characteristics and chemotherapy were obtained from surgical, pathological and oncological records. Recurrence and survival data were followed-up September 2007 (censoring date) using databases on hospital admission and the National Central Registry on death recording. The local research ethics committee has approved this study and for the samples to be used in research (KF01-201/03, 01-286965).

### Tumor samples

Archival tumor samples were collected from the pathological departments serving the surgical departments that referred cancer patients to Department of Oncology, Rigshospitalet. Out of 352 tumor specimens requested, 340 tumor specimens were accessible and contained representative tumor tissue, that were evaluated as to the type of carcinoma, degree of differentiation, perineural tumour growth and vascular invasion using one section from each block stained by routine heamatoxylin and eosin (HE) staining.

### Microsatellite analysis

Guided by microscopic examination of the HE stained slide, areas with at least 50% tumor cells present were grossly dissected from four 10 μm sections mounted onto glass slides, and DNA was isolated and prepared for microsatellite analysis. Microsatellite instability was determined in tumor DNA without the need for matching normal tissue DNA [23] using the highly specific panel of five quasimonomorphic mononucleotide repeats NR-21, NR-22, NR-24, BAT-25, and BAT-26 [23,24] in a pentaplex polymerase chain reaction as previously described [25]. For the purpose of prognostic evaluation microsatellite status was categorized as MSS (stable) versus MSI (instable) whether having instability in no (0) or more (≥1) markers, respectively.

### Immunohistochemistry

From the tissue specimens 2 mm cylinders were punched out and collected in microarray paraffin blocks each containing 40 samples. Tissue from normal kidney, liver and lymph node was included in the tissue arrays serving as reading frame and controls.

Sections of 4 μm were cut from the arrays and deparaffinized in xylene and rehydrated. Antigen retrieval was performed by immersing the slides in a 10 mM citrate buffer (pH 6 for hMLH1 and pH 7 for hMSH2, hMSH6, and hPMS2), and heating them in a microwave oven for 30 min at 95°C. Endogenous peroxidase was quenched by incubation of samples with 0.3% hydrogen peroxide in methanol. After washing in phosphate buffered saline (PBS), the sections were placed in 20% normal goat serum in PBS for 20 minutes to reduce non-specific staining. Sections were then incubated with monoclonal antibodies raised against hMLH1 (clone G168-15, 1:50 dilution; BD PharMingen, San Diego, CA), hMSH2 (clone NA27, 1:30 dilution; Oncogene Research Products, Darmstadt, Germany), hMSH6 (clone 44, 1:200 dilution; BD Transduction Laboratories), or PMS2 (clone 37, 1:250 dilution; BD PharMingen) for 1 hour at room temperature. TS and DPD stainings were done as previously described [20]. Subsequently, visualization was performed using the DAKO EnVision Duallink technique (DAKO, Glostrup, Denmark) according to the manufacturer's instruction and using diaminobenzidine as a chromogen.

Immunohistochemical staining of TS and DPD was assessed semi-quantitatively. TS staining intensity was scored (0–3) using the scale 0: no staining, 1: weak staining, 2: moderate staining, and 3: intense staining. Tumour cells with the highest DPD intensity were evaluated and assigned a score (0–3) using the scale 0: no staining, 1: faint ambiguous staining, 2: partly staining of cell membranes, 3: staining of cell membranes and cytoplasm. MMR proteins were scored as either no or positive staining. During loss of expression of a MMR protein the heterodimer may degrade too resulting in distinct patterns of absence of nuclear staining for each of the proteins hMLH1 (hMLH1, hPMS2), hMSH2 (hMSH2, hMSH6), and hMSH6 (hMSH6). Definite nuclear staining of adjacent non-tumor cells (e.g. lymphocytes, fibroblasts and endothelial cells) in the tissue array served as an internal positive control. Negative controls were performed by omitting the primary antibodies and by application of an isotype-matched non-reactive immunoglobulin in each staining run. Tissue specimens were analysed blinded to all other analyses and clinical information.

### Statistics

The relationships between tumor microsatellite status and clinicopathologic features were analyzed using Chi-square test or Mann-Whitney U-test, as appropriate. Survival time was calculated from the time point of the complete resection of the tumor. The outcome variables were recurrence free survival (RFS), defined as time to relapse of primary disease or death, whichever occurred first, and death from any cause for overall survival (OS). Distributions of RFS and OS were displayed using Kaplan-Meier methodology, and univariate survival distributions were compared using the log-rank test. Cox proportional hazard modeling was used to evaluate the association of RFS and OS with clinicopathological characteristics and MSI and MMR status. All candidate prognostic variables were initially entered into the model, and non-significant (P > 0.1) variables were subsequently rejected (step-down variable selection). Graphical methods were used to ascertain underlying model assumptions as proportional hazards. The interaction between TS or DPD expression and microsatellite status with outcome was evaluated in a Cox model. Multivariate logistic regression was used to test for the association of MSI status with the independent variables TS, DPD and clinicopathological characteristics. Kappa statistics was performed for comparison of MSI and MMR analyses. Two-sided P < 0.05 was considered statistically significant. Statistics was performed with Statistica software (Statsoft Inc. Tulsa, OK, USA).

## Results

### Clinicopathology, microsatellite instability and mismatch repair protein expression

Microsatellite status was assessed in 311 (92%) of 340 tumors. In the remainders analyses failed for technical reasons partly because of DNA degradation in tumor necrosis. In the microsatellite study sample 43 (13.8%) tumors had MSI. In the MSI subset the frequencies of mutated microsatellite loci were for NR-21 41 (95.3%), NR-22 38 (88.4%), NR-24 28 (65.1%), BAT-25 42 (97.7%), and BAT-26 42 (97.7%), respectively. MSI tumors had instability in at least three and in most cases four markers, while MSS tumors had none.

Expression of mismatch repair proteins hMLH, hMSH2, hMSH6 and hPMS2 was assessed in 306 (90%) tumor specimens using immunohistochemistry. The analysis failed in the remaining archival samples mainly because of loss of tissue cylinders during sectioning of arrays. Absence of repair protein expression was stated in 52 (17.0%) tumors. The coincidental absence of nuclear staining of a repair protein and its heterodimer indicated that loss of expression had primarily occurred for hMLH1 in 39 (12.7%), hMSH2 in 5 (1.6%), and for hMSH6 in 8 (2.6%) tumors.

Clinical and pathological characteristics according to microsatellite status are shown in Table 1. MSI tumors had a bimodal age distribution with significantly more elderly patients (P = 0.02) and few younger patients. Tumors with MSI were poorly differentiated (P = 0.001). MSI tumors were mainly located proximally to the splenic flexure in ascending and transverse colon (P = 0.001), and had minor risk of ileus at resection (P = 0.01). There were no statistically significant differences (P > 0.05) according to microsatellite status regarding gender, stage, vascular tumor invasion, perineural tumor invasion, or tumors complicated by perforation.

**Table 1:** Clinicopathological characteristics and tumor biomarker score according to microsatellite status.

	MSI* n = 43		MSS* n = 268		P
			
	No.	(%)	No.	(%)	
Gender					
Male	21	(49)	138	(51)	0.9
Female	22	(51)	130	(49)	
Age					
<70	28	(65)	217	(81)	**0.02**
≥70	15	(35)	51	(19)	
Tumor site					
Proximal colon	36	(84)	68	(25)	**0.001**
Distal colon	6	(14)	145	(54)	
Rectum	1	(2)	55	(21)	
Stage					
II	6	(14)	26	(10)	0.4
III	34	(79)	210	(78)	
IV	3	(7)	32	(12)	
Differentiation grade (WHO)					
Well	5	(12)	88	(33)	**0.001**
Intermediate	7	(16)	121	(45)	
Poor	31	(72)	59	(22)	
Perineural tumor invasion					
+	5	(12)	53	(20)	0.5
-	19	(44)	137	(51)	
not assessed	19	(44)	78	(29)	
Vascular tumor invasion					
V0	18	(42)	142	(53)	0.8
V1	8	(19)	58	(22)	
Vx	17	(39)	68	(25)	
Intestinal perforation at resection					
yes	2	(5)	29	(11)	0.2
no	41	(95)	239	(89)	
Bowel obstruction before resection					
yes	1	(2)	44	(16)	**0.01**
no	42	(98)	224	(84)	
Thymidylate synthase level					
Low	12	(28)	218	(81)	**0.001**
High	30	(70)	47	(18)	
not assessed	1	(2)	3	(1)	
Dihydropyrimidine dehydrogenase level					
Low	21	(49)	163	(61)	0.1
High	21	(49)	97	(36)	
not assessed	1	(2)	8	(3)	

*instable (MSI) or stable (MSS) microsatellites

### Microsatellite instability and mismatch repair protein expression

Compared to MSI status as the standard the immunohistochemical analyses of MMR proteins had a sensitivity of 0.95, a specificity of 0.93, a positive predictive value of 0.67, and a negative predictive value of 0.99, that resulted in an overall accuracy of 0.93. Correspondence between MSI readings and immunohistochemical analyses led to a Kappa value of 0.75.

### Disease recurrence and survival

During follow up (median 6.1 years; range 4.1–11.3 years) 121 patients (36%) had documented recurrent disease and 153 patients (45%) have expired. Median overall survival was 9.5 years, and the 5 year survival rate was 62%.

### Microsatellite instability, mismatch repair protein expression and outcome

**Figure 1:**
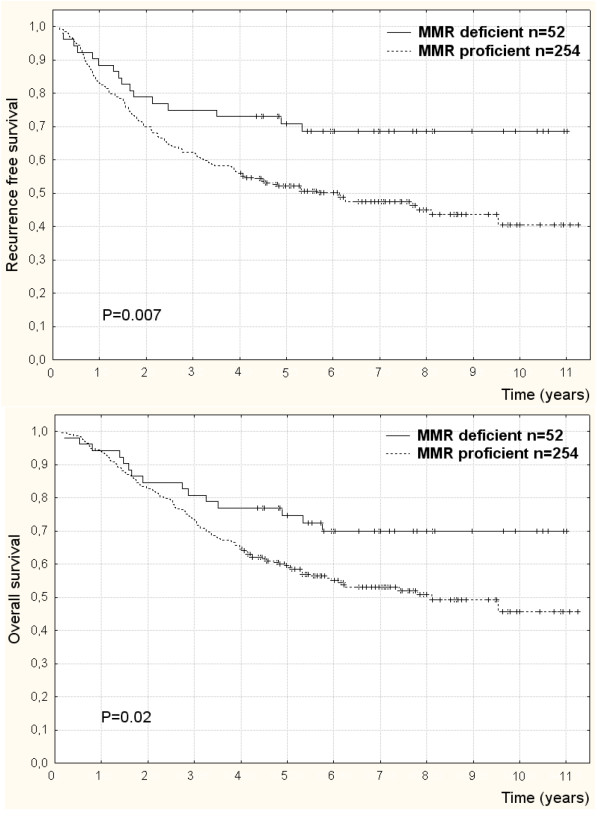
**Recurrence free survival (a) and overall survival (b) according to mismatch repair deficiency from loss of either hMSH2, hMSH6, or hMLH1 in colorectal cancers of patients completely resected and adjuvantly treated with 5-fluorouracil**. Censored data (+).

In univariate analyses of outcome according to MMR status stratified by disease stage (Table 2), patients having MMR deficient compared to MMR proficient tumors had significantly lower risk of recurrence (HR = 0.5; 95% CI: 0.3–0.9; P = 0.006) (Figure 1a) and death (HR = 0.6; 95% CI: 0.3–1.0; P = 0.04) (Figure 1b). In multivariate analysis of outcome MMR deficient compared to MMR proficient tumors were significantly associated with lower risk of recurrence (HR = 0.4; 95% CI: 0.3–0.8 P = 0.003) and death (HR = 0.5; 95% CI: 0.3–0.9; P = 0.02), when controlling for the influence of other independent predictors of recurrence; disease stage (P = 0.001), perineural invasion (P = 0.05), and ileus (P = 0.0002).

**Table 2:** Outcome according to microsatellite instability and mismatch repair deficiency stratified by disease stage.

		Recurrence free survival				Overall survival			
			
	No.	Events	Hazard ratio (95% CI)		P	Events	Hazard ratio (95% CI)		P
Microsatellite status*									
All stages	311	113	0.4	0.2–0.7	**0.002**	138	0.5	0.2–0.9	**0.02**
II	32	8	-	-	-	13	-	-	-
III	244	81	0.5	0.3–0.9	**0.04**	101	0.6	0.3–1.2	0.2
IV	35	24	-	-	-	24	-	-	-
Mismatch repair competence*									
All stages	306	149	0.5	0.3–0.9	**0.006**	132	0.6	0.3–1.0	**0.04**
II	36	14	-	-	-	13	-	-	-
III	241	112	0.6	0.4–1.0	**0.04**	100	0.7	0.4–1.2	0.2
IV	29	23	-	-	-	19	-	-	-

*hazard ratios for instable (MSI) relative to stable (MSS) microsatellites, and mismatch repair deficient relative to proficient tumors.

**Figure 2:**
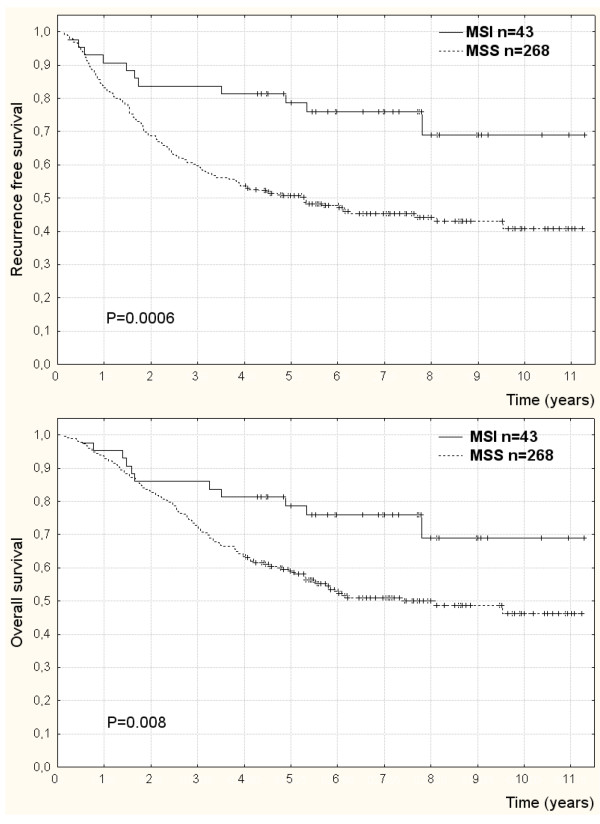
**Recurrence free survival (a) and overall survival (b) by instable (MSI) and stable (MSS) microsatellites in tumors of colorectal cancer patients completely resected and adjuvantly treated with chemotherapy**. Censored data (+).

**Figure 3:**
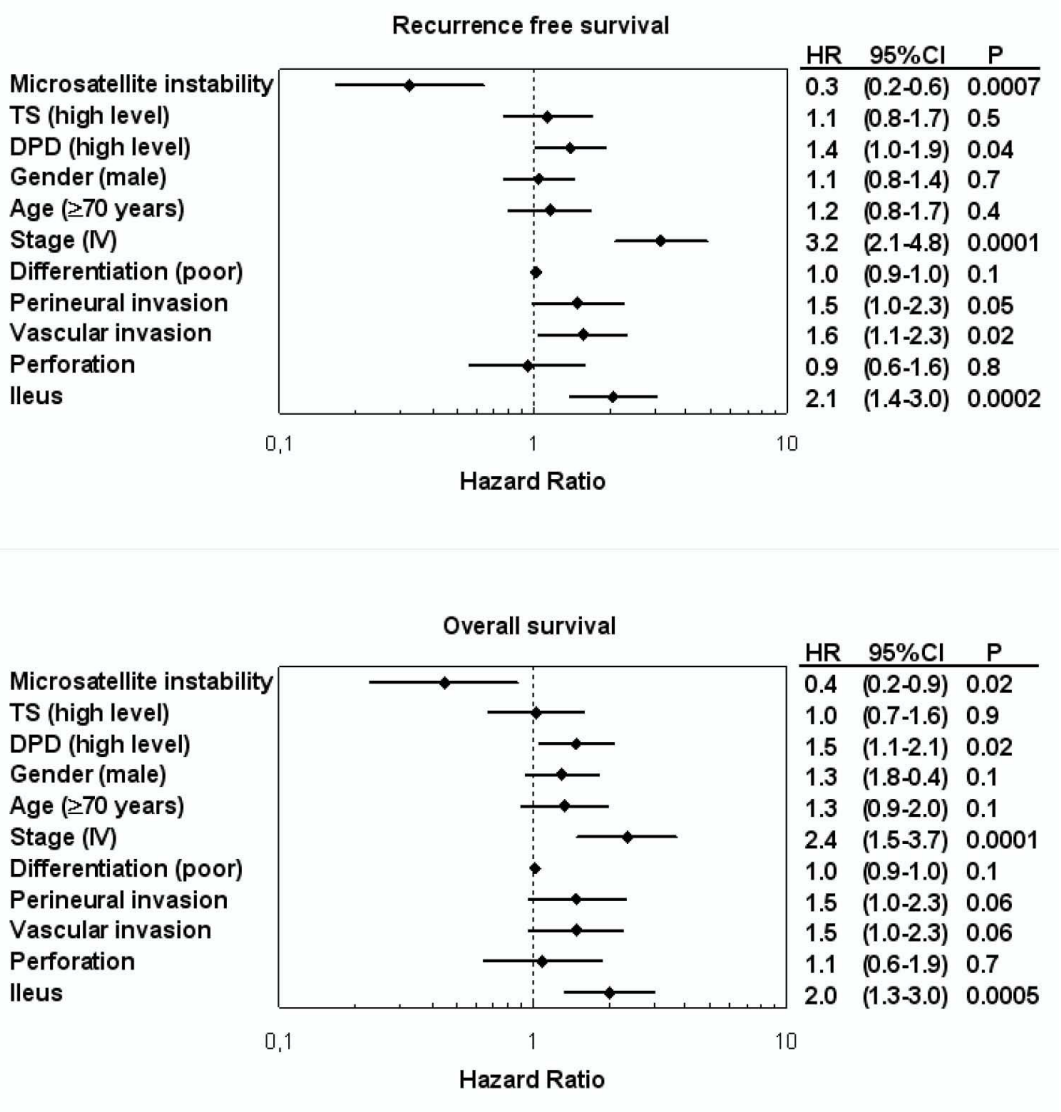
**Forest plots displaying multivariate Cox analysis of variables prognostic to recurrence free survival and overall survival following complete resection of colorectal cancer and adjuvant chemotherapy**. The prognostic variables included clinicopathological characteristics, tumor microsatellite status, expression of thymidylate synthase (TS) and dihydropyrimidine dehydrogenase (DPD).

Similarly, in univariate analyses of outcome according to microsatellite status stratified by disease stage (Table 2), patients with MSI compared to MSS tumors had significantly lower risk of recurrence (HR = 0.4; 95%CI: 0.2–0.7; P = 0.002) (Figure 2a) and death (HR = 0.5; 95% CI: 0.2–0.9; P = 0.02) (Figure 2b). Also in multivariate analysis of outcome MSI compared to MSS tumor patients had significantly lower risk of recurrence (HR = 0.3; 95% CI: 0.2–0.7; P = 0.0007) (Figure 3a) and death (HR = 0.4; 95% CI: 0.2–0.9; P = 0.02) (Figure 3b) adjusted for the prognostic influence of disease stage (P = 0.0001), vascular invasion (P = 0.02), perineural invasion (P = 0.05), and ileus (P = 0.0002).

### Microsatellite instability and association with TS and DPD expression

The distribution of biomarkers according to microsatellite status (Table 1) indicated a direct relationship between MSI and increasing TS staining intensity (P = 0.001), while there was no evidence of an association with DPD staining intensity (P = 0.1). Similar results applied to the relationship between MMR status and expression of TS (P = 0.0001) and DPD (P = 0.3).

Logistic regression testing relating MSI status and clinicopathological variables to the value of biomarkers as dependent variables revealed statistically significant association with increasing TS expression (global P = 0.0001), and non-significant association with DPD expression (P = 0.4).

Tests for interaction between biomarker expression and MSI status to predict outcome, using Cox analysis, were statistically significant for TS for recurrence (P = 0.002) and overall survival (P = 0.02), and non significant for DPD for recurrence (P = 0.5) and overall survival (P = 0.9). With adjustment for multiple comparisons of four interactions considered, involving two biomarkers and two outcomes, the test values for TS were statistically significant for recurrence (P = 0.01) and non significant for overall survival (P = 0.1).

## Discussion

In keeping with previous reports [2-4,6] and a meta-analysis [11] this study found a favourable outcome associated with MSI as compared to MSS of resected colorectal carcinomas stage II-IV in the adjuvant setting. These findings were based on uniform criteria for categorization of microsatellite instability using the NCI recommended reference panel of five loci [24]. In addition, characterization of mismatch repair competency by assessing expression of four main mismatch repair proteins corroborated these results. Others reported no prognostic significance of microsatellite instability in this setting, based on various criteria for microsatellite status [5,26-29].

Initial investigations into the predictive role of microsatellite instability showed similar improvement in outcome from adjuvant 5-fluorouracil therapy irrespective of microsatellite status of the resected adenocarcinomas [28,30]. Inadvertantly biased treatment groups for comparison may have accounted for this conclusion. In later reports improved outcome from adjuvant 5-fluorouracil in terms of reduced recurrence rate and better overall survival related to patients with microsatellite stable tumors only [2,5,12,29,31-33], whereas the subset having MSI cancers gained no similar beneficial effect from chemotherapy [11]. Hence the prevailing evidence suggests that 5-fluorouracil therapy should not be given to patients with MSI colorectal cancer.

On these premises the outcome according to microsatellite status in the present study can therefore be ascribed mainly to the biology of MSI colorectal cancer and to a lesser extent to antitumor response to 5-fluorouracil therapy. Other clinicopathological features of the MSI carcinomas may contribute to the better prognosis. Hence the minor risk of bowel obstruction associated with right sided tumors is an independent favourable prognostic variable. Moreover the generally lower staging at diagnosis is taken to indicate minor propensity of MSI tumors to metastasize [11,31,34,35]. Possibly the decreasing frequency of MSI by stage was not as evident in this cohort because of selection. According to the current treatment algorithm most stage II cancers are not referred for adjuvant chemotherapy unless they have additional poor prognostic factors.

In multivariate analysis the outcome of MSI tumor patients was independent of the TS and DPD levels, suggesting that differential expression of these enzymes could not account for the favourable natural history nor the resistance to chemotherapy [2,5,12,29,31-33].

The major part of an administered dose of 5-fluorouracil is catabolized by dihydropyrimidine dehydrogenase (DPD) into 5,6-dihydrofluorouracil, before it can be converted to the active metabolite fluoro-2'-deoxyuridine-monophosphate that irreversibly inhibits thymidylate synthase. Also the intracellular conversion to 5-fluorouridine and 5-fluoro-2'-deoxyuridine and corresponding phosphates for incorporation into RNA and DNA, respectively, have cytotoxic effect [13]. The similar magnitude of DPD expression regardless of tumor microsatellite status is consistent with MMR deficient cells accumulating 5-fluorouracil adducts in DNA [13], which is possibly tolerated due to inability to recognise adducts and to initiate apoptosis.

The correlation between high TS expression and microsatellite instability noticed in this and other studies [36,37] should be interpreted cautiously, as it may not explain prognostic and chemotherapeutic response characteristics of MSI and MMR deficient tumors. Accordingly high TS expression has generally been associated with early disease recurrence independently of chemotherapy [14-18,21], while also being related to improved outcome from adjuvant 5-fluorouracil treatment [14-19]. On the other hand, low TS expression has been related to low spontaneous recurrence rate [14-18,21], while indicating relative 5-fluorouracil resistance as well [14-19].

Unlike previous reports [5,38] the potential interaction between TS expression and microsatellite status with disease recurrence was based on a marginal direct relationship between TS level and outcome for patient with MSI tumors, which did not apply to the MSS subset. The low metastatic capacity [11,31,34,35] and high apoptotic index [39] of MSI tumors may counterbalance metabolic features otherwise linked to poor prognosis [14-18,21].

Earlier reports on the relationship between TS intensity and microsatellite instability have been conflicting [5,36-38,40-44] arguing either for a direct correlation [36,37] or no such association [5,38,41,42]. The discrepancy may partly relate to lack of consistency in criteria defining microsatellite instability, as some studies were based on single microsatellite marker [38,40] whereas others [5,36,44] applied more restricted criteria [24]. Also technical matters regarding immunohistochemical assessment of TS expression may have led to different results. While 24% of cases in this cohort had high TS score, the proportion in other studies has ranged between 19–77% of colorectal cancers in the adjuvant setting [45].

As prospective trials usually exclude elderly patients [5], also the age distributions of patient cohorts may have varied between studies. This raises the question whether high TS expression is confined to either inherited or sporadic MSI cancers that usually arise at an average age in the mid-forties or beyond the age of 70 years, respectively [46]. Difference in TS expression might be of significance to varying outcome of MSI tumors seen in the context of inherited repair deficiency [33].

Significantly higher TS score in tumors deficient of hMLH1 as compared to those deficient of hMSH2 or hMSH6, actually did support the notion that TS expression may vary according to the etiology of mismatch repair deficiency.

Though the pattern of microsatellite instability and resultant influence on gene deregulation may depend on the mechanism of mismatch repair deficiency, no causal connection can be deduced from the high TS expression found in MSI tumors. The fact that microsatellite instability not being involved in recombinant events leading to TS gene variability [40,43,44] suggests that the phenomenon is rather of secondary character. Thus the somewhat paradoxical mucinous histology and poor differentiation of MSI tumors [38,41] have metabolic traits implying higher intensity and diffuse pattern of TS expression [20].

Taken together there are no evidence to suggest direct influence of microsatellite instability on DPD or TS expression, nor that differential expression of these enzymes mediates the features for tumor biology or 5-fluorouracil resistance of MSI carcinomas.

The discrepancy in results obtained with the MSI and immunohistochemical methods may have various causes. The subsets analysed with either methods in this study is partly overlapping for technical reasons as indicated in results section. The most significant limitation of immunohistochemistry is the semiquantitative nature of immunostaining and the loss of sensitivity secondary to antigenic alterations caused by fixation procedures. Faint staining may be considered non-specific leading to higher sensitivity and lower specificity for defining MMR deficiency, or vice versa. Moreover a mutant protein product can be expressed and detected by immunohistochemistry. Conversely MMR protein mutations may occur in tumors displaying no MSI. These analytical shortcomings preclude that the methods may unambiguously reflect each other.

## Conclusion

Microsatellite instability due to MMR deficiency is one of the main biomarkers in colorectal cancer, as it not only indicates the pathogenesis, but also provides information on prognosis and prediction of response to chemotherapy. Future investigations into gene targets for microsatellite instability-driven deregulation may clarify the molecular foundation for the distinct clinicopathological characteristics of MSI carcinomas.

## Abbreviations

MSI: Microsatellite instability; MSS: microsatellite stability; MMR: mismatch repair; TS: thymidylate synthase; DPD: dihydropyrimidine dehydrogenase.

## Competing interests

The authors declare that they have no competing interests.

## Authors' contributions

SAJ and JBS conceived and planned the study. SAJ collected and integrated the clinical data. BV collected the tumor specimens from the departments of pathology. BV conducted the immunohistochemical analyses, scoring and interpretations. MK performed the microsatellite analyses, readings and interpretations. SAJ performed data analyses, and made the curve plots and the statistics. SAJ and JBS interpreted the results and SAJ wrote the manuscript draft. All authors critically read through and contributed to the revision of the manuscript draft. All authors approved the final manuscript.

## Pre-publication history

The pre-publication history for this paper can be accessed here:

http://www.biomedcentral.com/1471-2407/9/25/prepub
